# Parkinson’s disease functional movement battery a comprehensive test set to evaluate of motor abilities in persons with Parkinson’s disease

**DOI:** 10.1038/s41598-025-96594-3

**Published:** 2025-04-08

**Authors:** Bouwien Smits-Engelsman, Jacques Duysens

**Affiliations:** 1 Physical Activity, Sport and Recreation, Faculty Health Sciences, North-West University, Potchefstroom, South Africa; 2 Motor Control Laboratory, Movement Control and Neuroplasticity Research Group KU Leuven, Leuven, Belgium

**Keywords:** Parkinson, Manual dexterity, Balance, Locomotion, Structural validity, Hoehn & Yahr, Health care, Neurology

## Abstract

Clinicians require quantitative measures of functional movement to inform care decisions for persons with Parkinson’s disease (PWPs). To address this need, we developed the Parkinson’s Disease—Functional Movement Battery (PD-FUNC), which includes valid items from existing assessments and evaluates five key areas from the MDS-UPDRS-III: manual dexterity, functional strength, locomotion, static balance, and activities of daily living. This study assessed the PD-FUNC’s ability to distinguish between PWPs and healthy controls based on effect sizes and analyzed differences according to disease progression using years since diagnosis and MDS-UPDRS-III scores, as well as Hoehn & Yahr (H&Y) stages. The test was administered to 81 PD patients (H&Y stages 1–3) and 81 age-matched controls. All items, except grip force, discriminated effectively, with dexterity tasks showing the highest sensitivity (effect size r = 0.52–0.63). The PD-FUNC distinguished PD stages well, revealing early symptoms through dexterity tests (p = 0.001) and late-stage symptoms via dynamic stability tests. The PD-FUNC could provide a comprehensive assessment within 30–40 min and could be used to evaluate disease progression and medication effectiveness at home and in clinical settings.

## Introduction

The Movement Disorder Society-sponsored revision of the Unified Parkinson’s Disease Rating Scale (MDS-UPDRS)^1^ is considered the gold standard for evaluating motor symptoms in Parkinson’s disease. However, it has notable disadvantages, including subjective scoring, which necessitates highly trained examiners and is often time-consuming^2,3^. This highlights the need for objective, quantitative measures to assess functional movement capacity in persons with Parkinson’s (PWPs).

Previous efforts, such as the Timed Motor Test (TMT) battery, have provided initial steps toward this goal, demonstrating that the pegboard test is particularly sensitive for measuring dexterity in PWPs. However, the TMT lacks items that assess strength or balance and offers only rudimentary gait evaluations^2,3^. Alternatively, the upper limb Physiological Profile Assessment (PPA) was designed to evaluate arm function in PWPs^4,5^, but it does not address leg function, despite the existence of related assessments for older adults to estimate fall risk^6^.

While quantifying gait and balance deficits is crucial for evaluating disease progression, existing tests often fall short, with most clinical rating scales inadequately assessed for their psychometric properties^7^. Although various initiatives exist, they frequently lack comprehensive tests summarizing these various functions^8^.

To address these gaps, we propose a set of comprehensive yet simple, low-cost tests that quantitatively measure the fundamental domains outlined in the MDS-UPDRS- part II and III: manual dexterity (5 items, plus typing and handwriting), functional strength (4 items), locomotion (6 items), static balance stability (3 items for each leg), and activities of daily living (ADLs) (2 items). The Parkinson’s Disease Functional Movement Battery (PD-FUNC) incorporates elements from established protocols, including the Functional Strength Measurement (FSM)^9^, the Movement Assessment Battery for Children (MABC)^10,11^, SOS^12,13^, and the Performance and Fitness Test Battery (PERF-FIT)^14,15^. While these tests were primarily developed for children with motor coordination disorders, they include items applicable to PWPs.

Bloem et al.^7^ emphasized the necessity for simple tests suitable for prolonged assessments in patients’ home environments. The PD-FUNC has been applied in a home setting over several years, largely overcoming the limitations of previous assessments (see Appendix 2). Furthermore, the PD-FUNC can be learned quickly (within 2–3 h) and utilizes readily available tools. Longitudinal application of this battery in clinical practice could provide insights into disease progression, could potentially be used to measure the impact of medication, and facilitate therapeutic decision-making. Consistency in reported outcomes in PD research using the same tool will also enhance comparative research.

## Methods

### Study Design and Participants

Eighty-one persons with Parkinson’s (PWPs) participated in this study, matched as closely as possible for gender and age with a cohort of healthy controls. The median time since diagnosis was 3 years (range 0.5–29 years) and 68% of participants were diagnosed within the last 6 years. The median age at diagnosis was 67 (range: 39–87). No significant differences were found between the PD and control groups in age (p = 0.14), weight (p = 0.98), height (p = 0.73), or gender distribution (p = 0.60). However, the Mini-Mental State Examination (MMSE)^16^ scores differed significantly (z =  − 2.44, p = 0.015) (better range of scores in controls; see Table 1 for details). Among the 81 PWPs, 26 (32%) were classified as Hoehn & Yahr stage 1, 33 (41%) as stage 2, and 22 (27%) as stage 3; 60 participants (74%) were male and 21 (26%) female (see Table 2 for details). Notably, one patient was recently diagnosed and classified as H&Y stage 3. Almost all PWPs were on levodopa therapy, often in combination with dopamine agonists, and participants did not alter their medication schedules for testing. All participants completed the Physical Activity Readiness Questionnaire (PAR-Q)^17^, and if any questions were answered positively, their general practitioner was consulted to confirm that participation was safe. All participants signed a written consent. Ethical approval for this study was obtained from CMO Radboud UMC (file number 2022–15731).

**Table 1:** Demographic data for the PD group (n = 81) and the control group (n = 81).

Variable	PD Mean (SD)	PD Median (IQR)	Controls Mean (SD)	Controls Median (IQR)
Age	71.3 (8.2)	73 (67–78)	70.2 (9.0)	68 (63–76)
BMI	25.6 (3.3)	26 (23–27.5)	26.0 (3.4)	25 (24–28)
MMSE*	27.9 (1.9)	28 (27–30)	28.7(1.6)	29 (28–30)

	Number	Percentage	Number	Percentage
Male	60	74.1	57	70.4
Female	21	25.9	24	29.6

*Significantly different z -2.44, p = 0.015.

SD: Standard Deviation; IQR: Interquartile range; BMI: Body Mass Index; MMSE: Mini Mental State Examination.

**Table 2:** Demographic data for the individuals with Parkinson’s disease per Hoehn & Yahr stage.

Variable	H&Y1 (n = 26) Median (IQR)	H&Y2 (n = 33) Median (IQR)	H&Y3 (n = 22) Median (IQR)
Age	71 (60–76)	70 (66.5–74.5)	77 (73–80)
BMI	25.8 (23–26.6)	26.3 (23–27.8)	26.0 (24.4–28)
MMSE	29 (28–30)	28 (27–30)	27(25–29)
Years since diagnosis	2 (1–3.3)	4 (2–10)	4 (2–6)

	Number	Number	Number
Male	16	27	17
Female	10	6	5

IQR: Interquartile range; BMI: Body Mass Index; MMSE: Mini Mental State Examination.

### Procedures

Testing was conducted at physical therapy institutions or at the patients’ homes by twelve physical therapists as part of their training in master’s level geriatric physical therapy. Most therapists were affiliated with Parkinsonnet, a healthcare network specializing in care for PWPs. They received additional training on the PD-FUNC lasting 2–3 h. Following a review of the manual, all items were demonstrated, and scoring criteria were discussed. Each therapist piloted the administration of the tests with at least two individuals before data collection, and any questions about instructions or scoring were addressed online.

### Materials

In addition to demographic and Parkinson’s disease history, the following assessments were administered: MDS-UPDRS and PD-FUNC.

### MDS-UPDRS

The MDS-UPDRS consists of four parts^1^. Part I, concerning non-motor experiences of daily living, was deemed irrelevant for the current motor skill evaluation and was not used. Part II addresses motor experiences of daily living with 13 patient-reported items, while Part III, the motor examination, includes 18 items evaluated by a physical therapist (33 scores in total). Part IV assesses motor complications, focusing on dyskinesia and fluctuations through six items. Each item is scored from 0 (normal) to 4 (severe). The scoring definitions are as follows: “Slight” (1) indicates low-frequency or intensity symptoms that do not impact function; “Mild” (2) refers to symptoms causing a modest functional impact; “Moderate” (3) indicates symptoms that significantly impact but do not prevent function; and “Severe” (4) refers to symptoms that completely prevent function.

### Parkinson’s Disease Functional Movement Battery (PD-FUNC)

The PD-FUNC was constructed following the piloting of various items and includes 5 items for manual dexterity (Pegboard, Lace board, Tracing, Typing, Writing), 3 items for functional strength (Grip strength, Sit-to-stand, Lifting a 5 kg box), 4 items for locomotion (3 methods of traversing a 4-m agility ladder, rotation on the spot), 3 items for stability (2- and 1-legged stance), and 4 ADL activities (buttoning a shirt, putting on trousers, putting on socks, turning on a mat). A detailed description of each item and materials needed is provided in Appendix 1. The ADL tasks are not included in the quantitative analysis due to variability in clothing among participants; instead, they are qualitatively assessed and videotaped for individual follow-up. Standardization of clothing for group comparisons proved impractical. Moreover, some earlier tried-out balance tests (standing on a small balance beam) had to be excluded due to difficulty for the PWP population. The entire testing process in the retained items takes approximately 30–40 min, depending on the motor capabilities of the PWP.

### Statistics

Data distribution was assessed, and the choice of statistics was adapted accordingly. Due to the small sample size per subgroup, all outcomes were tested non-parametrically; both means (SD) and medians (Range) are reported of the described variables, for comparability to other studies.

A two-group comparison (Mann–Whitney U-test) was conducted to identify items which were significantly different between PWPs and controls. Effect sizes were calculated using the standardized test statistic z from the Mann–Whitney U-Test divided by the square root of the number of pairs, with effect size interpretations as follows: r < 0.3 (small), 0.3 ≥ r < 0.5 (medium), and r ≥ 0.5 (large)^18^.

Subsequently, a non-parametric four-group comparison (controls and three H&Y stages) was performed using the Kruskal–Wallis test with post hoc tests, Bonferroni correction was applied for multiple comparisons. Although the group sizes for this subgroup analysis were small, this analysis provided insights into differences in specific items across disease severity levels. To standardize outcomes for visualization, data were transformed into z-scores (mean of 0 and standard deviation of 1).

Spearman rank-order correlation coefficients were calculated between years since diagnosis, Part II and III of the UPDRS and the PD-FUNC items.

A power analysis indicated that a total sample size of 77 per group was needed for detecting a medium effect size (r ≤ 0.30) difference with 95% power at an alpha of 0.05, resulting in a 66.4% probability that a randomly sampled score from controls would exceed a score from PWPs.

## Results

### Discriminating PD from controls

**Fig. 1:**
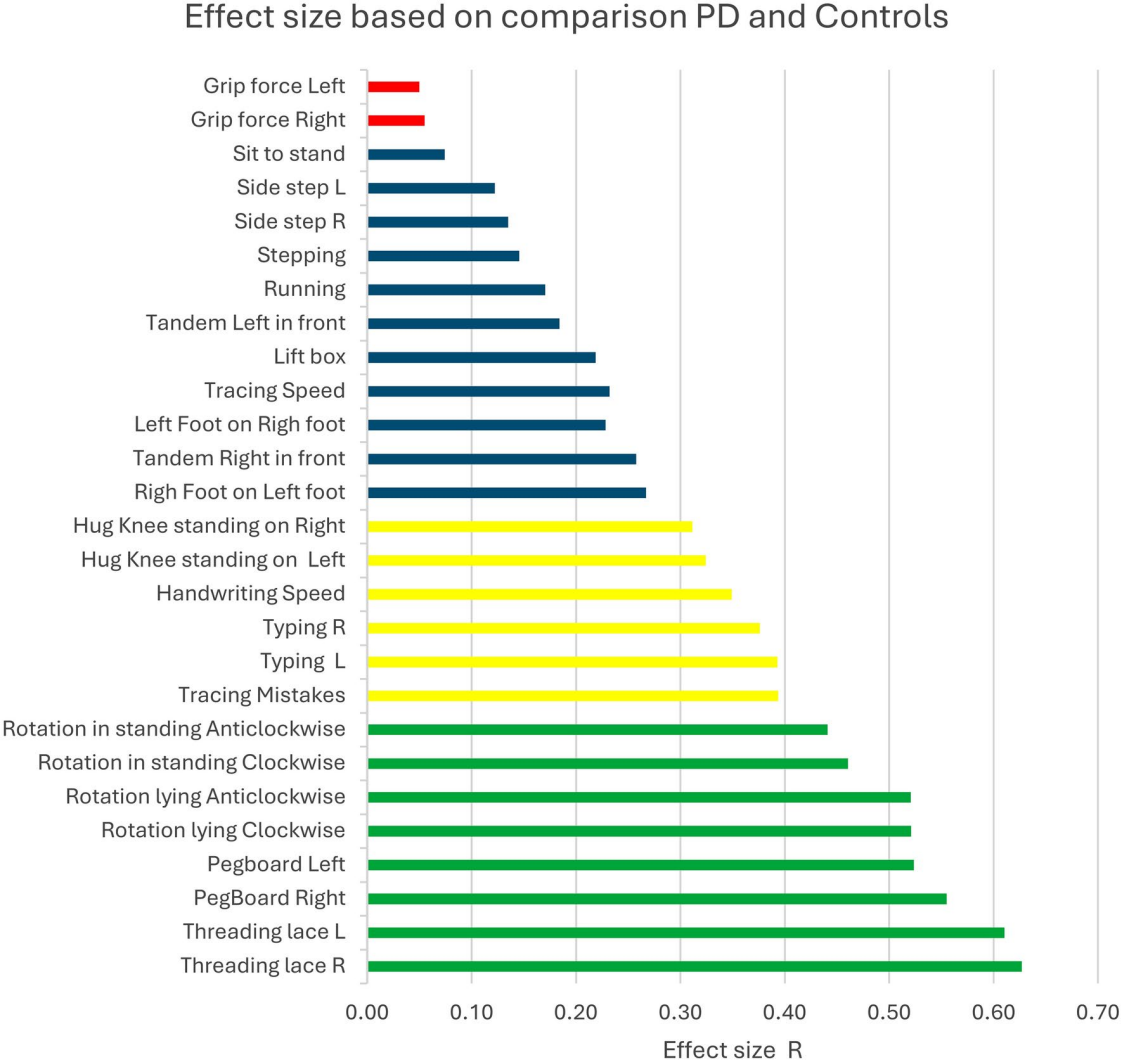
displays the magnitude and practical significance of the observed differences between the groups. Large effect sizes (r > 0.5) are colored green, medium effect sizes (≥ 0.3 and r < 0.5) are in yellow, and small effect sizes (r < 0.3) in blue. The difference for grip force between the groups was not significant and is represented in red.

All items of the Parkinson’s Disease Functional Movement Battery (PD-FUNC) effectively discriminated between persons with Parkinson’s disease (PWPs) and controls, with the exception of grip force. Ranked effect sizes are illustrated in Fig. 1. Items demonstrating large effect sizes, specifically fine motor and rotational tasks, were highlighted in green.

### Discrimination between levels of disease severity

**Fig. 2:**
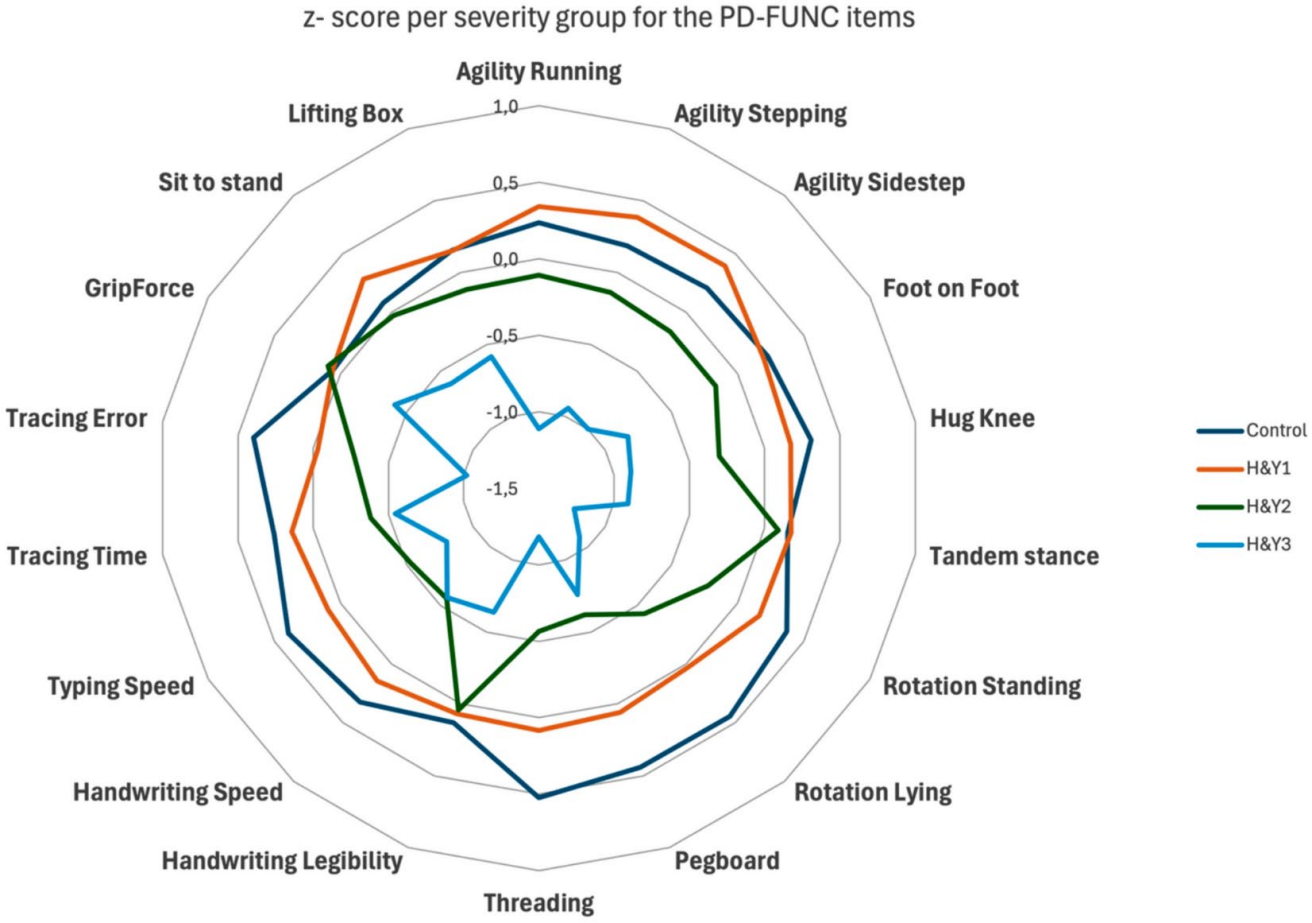
Depicts the item scores across severity groups in a radar chart using z-scores. The distance between the concentric circles indicates half a standard deviation. Notably, the Pegboard and Threading Lace tasks were sensitive measures for detecting differences between controls and across the various stages of PD. Conversely, for grip force scores only H&Y3 is different from controls.

Further differences were evaluated across the four severity levels (Control, H&Y 1, H&Y 2, and H&Y 3). The main group effects per item and post hoc pairwise comparisons are summarized in Table 3 and visualized in Fig. 2. Items sensitive to early detection (showing differences between Control and H&Y 1) were primarily dexterity tests, indicating that locomotion and balance are not yet affected at this early stage. In the comparison between Control and H&Y 2, typing and pen-and-paper tasks showed deterioration, while handwriting legibility remained preserved. Additionally, balance became more compromised. Comparisons between Control and H&Y 3 revealed significant differences in locomotion and a reduction in strength.

**Table 3:**
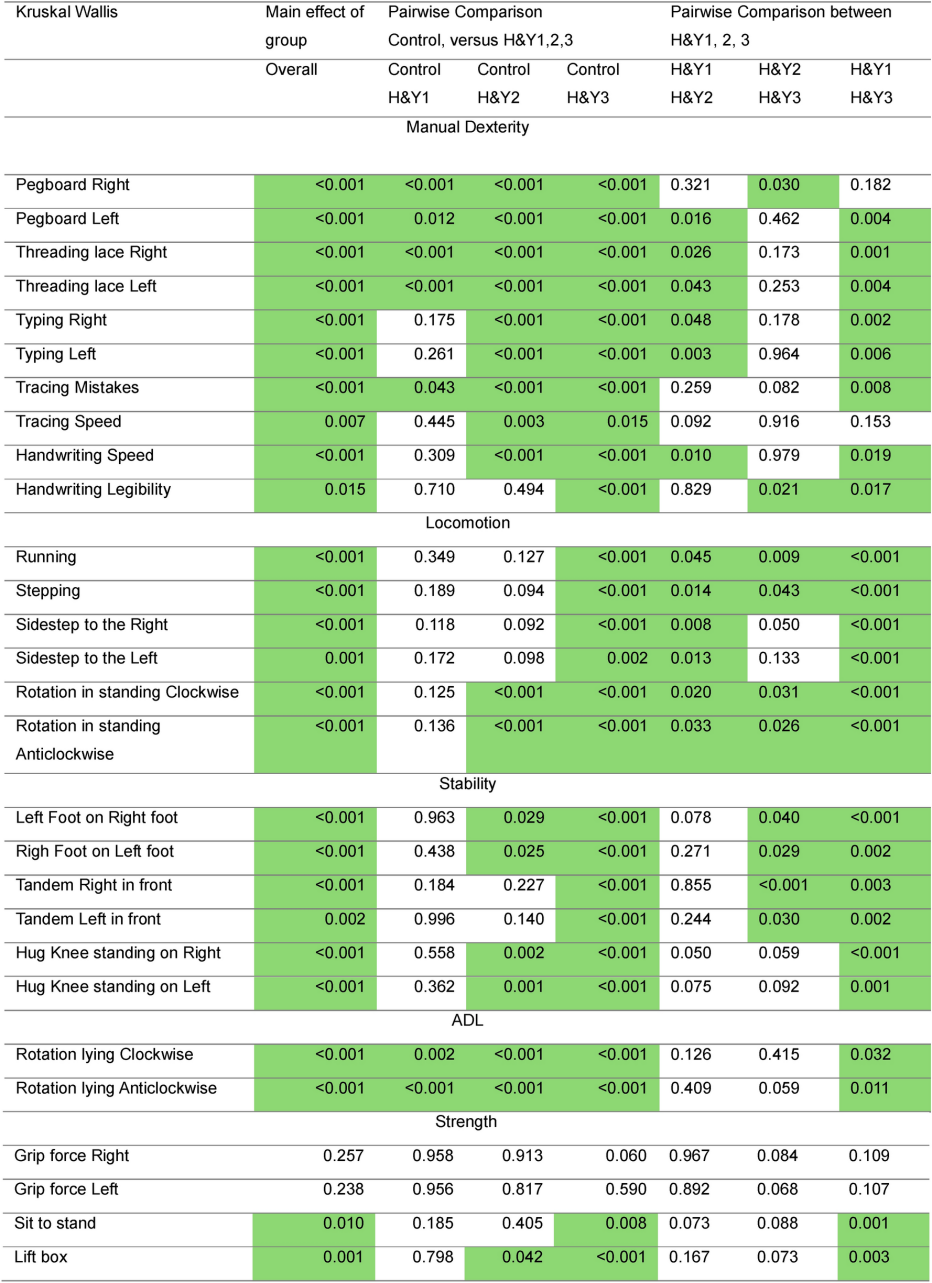
Provides an overview of the statistics from the Kruskal–Wallis test and post hoc pairwise comparisons. Items with pairwise comparison p-values < 0.05 are colored green.

### Association between instruments

Lastly, correlations were calculated between years since diagnosis, Part II and III of the UPDRS and PD-FUNC items, using data of all PWPs. For this analysis, means for bilateral items were used to limit the number of comparisons. As can be seen in Table 4 the association between PD-FUNC items and the UPDRS is mostly low or moderate and insignificant for stability. Fine motor items are the most frequently related to the UPDRS and are moderately related to years since diagnosis.

**Table 4:** Spearman correlation of the PD-FUNC items (means for bilateral items) with UPDRS Part II and III and Years since diagnosis (n = 81).

Variables	Part II UPDRS	Part III UPDRS	Years since diagnosis
Part II UPDRS	1.00	0.53**	0.16
Part III UPDRS	0.53**	1.00	0.25*
Years since diagnosis	0.16	0.25*	1.00
Manual Dexterity			
Peg Board	0.22*	0.32**	0.42**
Threading lace	0.35**	0.39**	0.30**
Typing (-)	0.30**	0.21	0.34**
Tracing Mistakes	0.22*	0.22*	0.36**
Tracing Speed	0.16	0.10	0.24*
Handwriting Speed (-)	0.40**	0.21	0.39**
Handwriting Legibility	0.22	0.39**	0.18
Locomotion			
Running	0.39**	0.28*	0.06
Stepping	0.40**	0.24*	0.03
Sidestep	0.39**	0.16	0.01
Rotation in standing	0.36**	0.23*	0.09
Stability			
Foot on foot (-)	0.14	0.19	0.05
Tandem (-)	0.19	0.18	0.04
Hug Knee (-)	0.14	0.16	0.08
ADL			
Rotation lying	0.13	0.20	0.21
Strength			
Grip force (-)	0.02	0.02	0.00
Sit to stand (-)	0.24*	0.19	0.03
Lift box (-)	0.21	0.12	0.00

Values in bolt represent moderate correlations (0.3 ≥ r < 0.5).

*p < 0.05 **P < 0.01, (-) negative correlations.

## Discussion

The aim of the present study was to evaluate whether a new set of motor tests (the PD-FUNC) could detect differences between individuals with Parkinson’s disease and healthy controls, considering disease progression. Unlike subjective questionnaires, quantitative methods can provide a more objective measure. One of the key findings is that nearly all test items could effectively discriminate between groups. Notably, tests which distinguished all three stages of the Hoehn and Yahr (H&Y) scale included pegboard, threading lace, and turning while lying on the floor. These assessments are critical for the early detection of PD, as they revealed significant differences even between controls and H&Y stage 1 participants.

### Manual dexterity

The most discriminating tests were those measuring dexterity. Early stages of PD can significantly impair the ability to grasp and manipulate objects. The association between fine motor skills and disease progression (years since diagnosis, UPDRS scores, H&Y stages) is particularly noteworthy. These skills may also predict cognitive decline and could serve as valuable screening tools^19,20^.

Our pegboard results corroborate the findings of Haaxma et al.^2,3^, who established that the pegboard test is a sensitive measure for evaluating motor dysfunction in early PD. This is consistent with current results, which demonstrated significant differences between controls and H&Y stage 1 participants. This also aligns with other studies by Alonso et al.^21^ and Earhart et al.^22^.

In addition to the pegboard test, we included items from the M-ABC with established validity (e.g., threading lace), which have seen limited application in Parkinson’s research^23^. Our results indicate that threading lace can serve as a new sensitive measure of bimanual dexterity.

We also incorporated a "one finger typing test" on a computer keyboard to assess bradykinesia, which aligns with other studies that utilized typing as an evaluation method^24,25^. This approach benefits from the ease of using a standard keyboard.

Handwriting was quantitatively assessed using a validated protocol (SOS)^13^ allowing for a more detailed evaluation of motor deficit severity. The fact that handwriting legibility remained preserved while handwriting speed deteriorated suggested that PWPs trade speed for legibility. Previous research has shown that handwriting assessments can detect changes post-intervention^26^ and may be useful for assessing medication efficacy^23^.

### Locomotion

The agility ladder tests (running and stepping) added valuable measures for evaluating disease progression concerning motor control, particularly dynamic balance and locomotor skills. Notably, in the sideways stepping task, H&Y stage 1 PWPs performed slightly better than controls, potentially due to visual cueing from the ladder rungs. Indeed, it is well known that visual cueing improves gait parameters in PWPs^27,28^. The increased challenge presented by the constraints of the ladder compared to simple walking on a flat surface emphasizes its utility.

The 360° turn emerged as the most effective test for eliciting freezing. While rotation in standing was normal at early stages, deficits became evident in later stages, making this test suitable for assessing fall risk and identifying freezing episodes in advanced PD^29,30^. This aligns with Zoetewei et al.^31^, who recommended combining turning with tests like the Timed Up and Go (TUG), which entails similar activities as incorporated in the PD-FUNC. The locomotion items showed the highest correlation, albeit moderate, of all PD-FUNC items with Part II UPDRS, which contains questions about Motor Aspects of Experiences of Daily Living filled out by the PWP.

### Stability

Regarding balance evaluation, static and dynamic balance tests (agility ladder) did not consistently show differences in the early stages of PD but were clearly affected by disease progression. Static balance tests revealed that stability was largely preserved in early stages (H&Y stage 1) but significantly deteriorated in later stages (H&Y stage 3), consistent with prior findings^6^.

Due to the high scores in the early stages, more challenging balance tasks were tried out during the pilot phase of the study. These included standing in tandem on a foam balance beam and two balance beam tasks from the MABC-2. While some participants with Parkinson’s disease (PWP) were able to mount the beams, regaining balance after falling or stepping off created hazardous situations and these tasks were therefore not included in the final item set.

### Activities of Daily Living (ADL)

The new test for rotation while lying on the floor was highly sensitive and ecologically valid, addressing a common issue for PWPs—turning in bed^32^. Difficulty with this task increased with disease progression, as indicated by significant differences between H&Y stage 1 and stage 3.

In earlier versions of the PD-FUNC, ecologically valid tests for ADL (e.g., shirt buttoning, putting on pants and socks) were included but ultimately excluded from quantitative analysis due to standardization challenges. Although some research has suggested the importance of these tasks^5^, they often present a too high level of dexterity that introduces a floor effect.

### Strength

An important negative finding was that grip strength was the only item showing no significant difference between PWPs and controls. This aligns with Alonso et al.^21^ but contrasts with Roberts et al.^31,33^, who found grip strength varied significantly with disease severity. The discrepancy may stem from differences in recruitment methods, as our participants were primarily recruited from physiotherapy practices, while Roberts et al. included a broader range of PWPs, some of whom may have had more advanced disease.

In general, the Stability, ADL and Strength items seem the least associated with duration of the disease as well as with the Motor Aspects of Experiences of Daily Living and Motor Examination parts of the MDS-UPDRS.

### Limitations and future research

This first clinical study using the PD-FUNC faced limitations, including a small sample size across the three severity groups and the potential for accidental findings due to the number of comparisons. Additionally, recruiting participants primarily from physical therapy practices specializing in care for PWPs may have led to the selection of well-trained individuals, which could affect the generalizability of findings to the broader PD population as differences may be larger between controls and PWP without receiving intervention.

The application of the PD-FUNC in larger cohorts of individuals with Parkinson’s disease should explore whether the PD-FUNC can detect clinically relevant changes, such as improvements in tasks mimicking daily activities, like turning in bed, manipulating small objects, or standing on one leg while putting on trousers.

In conclusion, the PD-FUNC could provide an objective measure complementing the MDS-UPDRS, enhancing treatment efficacy evaluation in clinical trials and quantifying changes in motor function over time, thereby guiding treatment decisions. Importantly, understanding motor function differences related to disease severity enables the development of personalized rehabilitation programs targeting specific deficits at various disease stages^34,35^. Longitudinal application of this battery in clinical practice could provide insights into disease progression and could potentially be used to measure the impact of medication and facilitate therapeutic decision-making.

## Supplementary Information

Supplementary Information 1.

Supplementary Information 2.

## Data Availability

All relevant data are within the manuscript and its supplementary files. Availability of data and materials. The full datasets presented in this article are not readily available because of Human Subjects’ protections. Requests to access the data sets should be directed to bouwiensmits@hotmail.com.

## Abbreviations

ADL: Activities of daily living
FSM: Functional Strength Measurement
H&Y: Hoehn & Yahr
MABC: Movement Assessment Battery for Children
MDS-UPDRS: Movement Disorder Society-sponsored revision of the Unified Parkinson’s Disease Rating Scale
MMSE: Mini-Mental State Examination
PAR-Q: Physical Activity Readiness Questionnaire
PERF-FIT: Performance and Fitness Test Battery
PD: Parkinson’s disease
PD-FUNC: Parkinson’s Disease Functional Movement Battery
PPA: Physiological Profile Assessment
PWPs: Persons with Parkinson’s
SOS: Systematische Opsporing van Schrijfmotorische problemen (Systematic Detection of writing problems)
TMT: Timed Motor Test
TUG: Timed Up and Go
UMC: University Medical Center
