# Supplementary material for: Parkinson’s disease functional movement battery a comprehensive test set to evaluate of motor abilities in persons with Parkinson’s disease
Source: Sci Rep. 2025 Apr 8;15:12035. doi: 10.1038/s41598-025-96594-3 (PMC11978789; doi:10.1038/s41598-025-96594-3)
Supplement: Supplementary file 1 — Supplementary Information 1. [file 41598_2025_96594_MOESM1_ESM.docx]

Parkinson’s Disease - Functional Movement Battery

(PD-FUNC)

Compilation of test items to be used to objectively evaluate functional movement capacity of patients with Parkinson’s disease or Parkinsonism

Bouwien Smits-Engelsman & Jacques Duysens

**Cape Town, Nijmegen 2020**

@ No changes or translations are allowed without permission from the authors.

For training videos please contact [JacquesDuysens@outlook.com](mailto:JacquesDuysens@outlook.com)

Table of Contents

[Overview of the test items 4](#_Toc188432764)

[Manual Dexterity Items 5](#_Toc188432765)

[1. Handwriting: SOS (Preferred Hand) 5](#_Toc188432766)

[2. Typing Speed (Left & Right) 7](#_Toc188432767)

[3. Pegboard (Turning Pegs, Left & Right) 8](#_Toc188432768)

[4. Threading Lace (Left & Right) 9](#_Toc188432769)

[5. Pen and Paper Trail (Preferred Hand) 10](#_Toc188432770)

[Functional Strength 11](#_Toc188432771)

[1. Sit to Stand 12](#_Toc188432772)

[2. Lifting Box 13](#_Toc188432773)

[3. Grip Strength (Left & Right) 14](#_Toc188432774)

[Walking with Directional Changes (Dynamic Balance, Freezing) 15](#_Toc188432775)

[1. Agility Ladder, Running 16](#_Toc188432776)

[2. Agility Ladder, Stepping 17](#_Toc188432777)

[3. Agility Ladder, Sideways (Left & Right) 18](#_Toc188432778)

[4. Rotation on Mat: Turn in Circle Clockwise and Anticlockwise 19](#_Toc188432779)

[Static Balance-Stability 20](#_Toc188432780)

[1. *Foot on foot* (15 seconds max) 20](#_Toc188432781)

[2. Tandem stance 20](#_Toc188432782)

[3. Knee hug 20](#_Toc188432783)

[Extra ADL Activities 21](#_Toc188432784)

[1. Putting on Pants 22](#_Toc188432785)

[2. Putting on Socks 23](#_Toc188432786)

[3. Buttoning Shirt 24](#_Toc188432787)

[4. Turning in Bed 25](#_Toc188432788)

# Overview of the test items

Manual Dexterity

1. Handwriting: SOS (Preferred hand)

2. Typing speed (Left & Right)

3. Pegboard (Left & Right)

4. Threading lace (Left & Right)

5. Pen and paper trail (Preferred hand)

Functional Strength

1. Sit to stand

2. Lifting box

3. Grip strength (Left & Right)

Walking with Directional Changes (Dynamic Balance, Freezing)

1. Agility ladder, Running

2. Agility ladder, Stepping

3. Agility ladder, Sideways (Left & Right)

4. Rotation on mat (clockwise and anticlockwise)

Static Balance-Stability

1. Foot on foot (Left & Right on top)

2. Tandem stance (Left & Right in front)

3. Knee hug (Left & Right stance leg)

ADL Activities (Timed and Videotaped for Qualitative Evaluation)

1. Putting on pants (sitting and/or standing)

2. Putting on socks (Left & Right)

3. Buttoning shirt

4. Turning in bed (on yoga mat, clockwise and anticlockwise)

## Manual Dexterity Items

### 1. Handwriting: SOS (Preferred Hand)

See instruction manual SOS-PD. B.C.M. Smits-Engelsman, E. Nackaerts, A. Nieuwboer

SOS-PD requires a manual, a sheet with the text to copy, general instructions, a score form, and a template for objectively assigning scores. The SOS has been developed to measure both quality (legibility) and quantity (number of letters) in handwritten text.

#### Materials:

- Unlined paper

- Copy sheet with text (available in Dutch, German, English, French, Arabic)

- Participant’s usual writing tool (sharp pencil, fountain pen, ballpoint pen, or fine liner)

- Stopwatch

- Transparent template for scoring

#### Instructions:

- Place your paper lengthwise on the table (if necessary, demonstrate) See figure S1.

- On the back of your sheet, write your name, age, gender, and whether you are right-handed or left-handed (the administrator should check this information for accuracy).

- Turn the page and start at the top, copying the text.

- Write the sentences in the same order as on the copying sheet.

- Use connected writing if that’s how you usually write; if not, write in your usual style.

- Write at your usual speed.

- Write as neatly and then as quickly as you can; keep writing until instructed to stop.

Figure S1: Item 1 copying text.


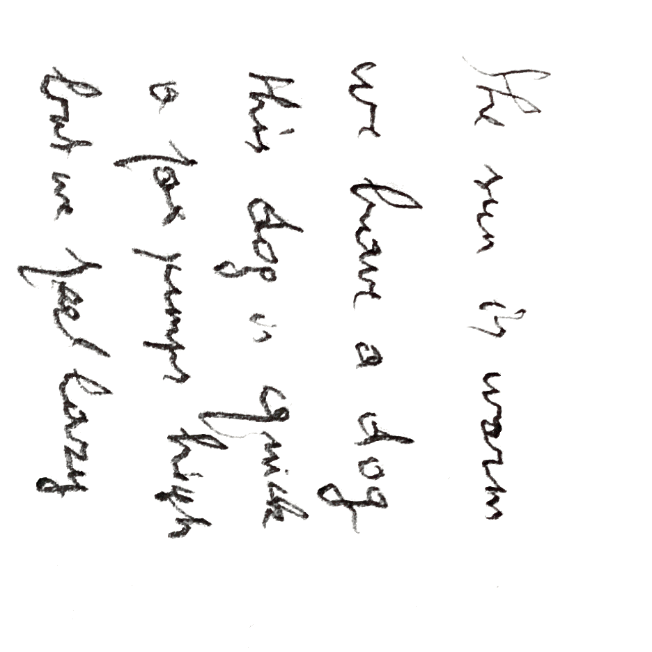

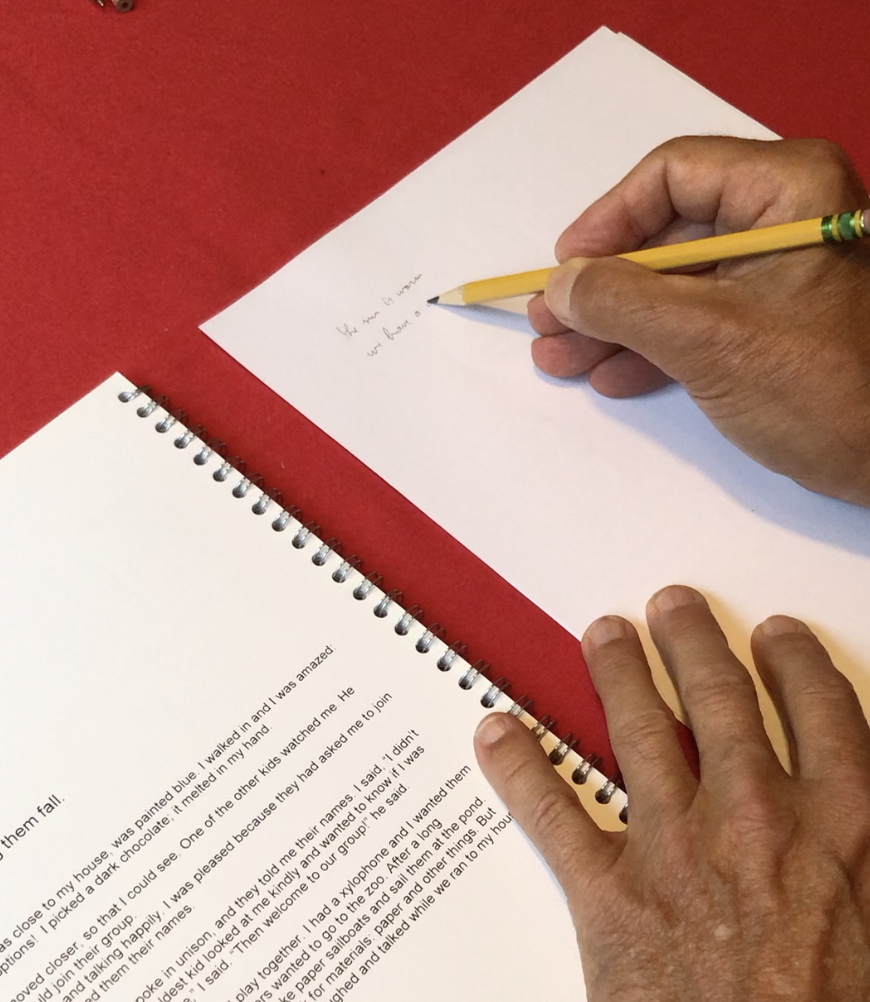


#### SOS-PD Score form

| Name of the patient: |  | | | Writing hand: | | |  | | |
| --- | --- | --- | --- | --- | --- | --- | --- | --- | --- |
| Date of Birth: |  | | | Hoehn & Yahr stage: | | |  | | |
| Test Date: |  | | | Male / Female | | |  | | |
| Name tester: |  | | |  | | |  | | |
| Line by line score the items with 0 (absent) or 1 (present) and on this base determine score for each item (with exception of item 4). When in doubt score 0 (absent). | | | | | | | | | |
| Choose either column: connected writing (SOS-PD-5i) or not-connected writing (SOS-PD-4i). | | | | | | | | | |
| **Item** | **Line 1** | **Line 2** | **Line 3** | | **Line 4** | **Line 5** | | **SOS-PD-5i** | **SOS-PD-4i** |
| Fluency |  |  |  | |  |  | |  |  |
| Transitions |  |  |  | |  |  | |  |  |
| Regularity letter size |  |  |  | |  |  | |  |  |
| Word spacing |  |  |  | |  |  | |  |  |
| Straight line |  |  |  | |  |  | |  |  |
| **Total Quality score** |  | | | | | | | | |
| Average letter size | ____________ mm | | | | | | | | |
| Speed | ____________ letters written in 5 minutes | | | | | | | | |

Writing problem based on SOS-PD: yes / no

Observation list

| Was tremor observed in writing hand? | Yes / No |
| --- | --- |
| Was tremor observed in paper holding hand? | Yes / No |
| Was upper limb freezing observed? | Yes / No |

### 2. Typing Speed (Left & Right)

Materials: PC and timer

#### Setup:

- Participant sits close to the table in front of the PC.

- Hands are positioned on the table next to the keyboard (see Figure S2).

#### Task:

- Left hand types alternately “a” and “s” with the index finger for 15 seconds.

- Right hand types alternately “k” and “l” with the index finger for 15 seconds.

Count the number of accurately typed letters (e.g., “aa” is counted as 1 letter).

Figure S2. Item 2 Typing start position.


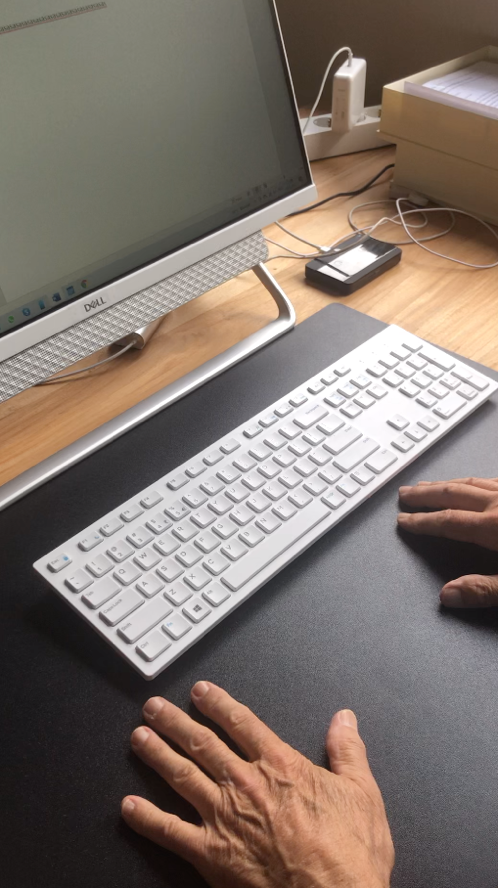


### 3. Pegboard (Turning Pegs, Left & Right)

See instruction manual MABC-2 age band 3 (Henderson, Sugden, Barnett 2007).

#### Setup:

The pegboard with pegs is positioned so that the same color is facing up (either all red or all yellow). See Figure S3

#### Instructions:

- Hands should be on both sides of the pegboard at the “Ready, Steady, Go” signal.

- Start the timer when hands leave the table.

- All pegs must be turned using finger manipulation and inserted again with the opposite color facing up.

- Record the time it takes to turn all the pegs. Stop the timer when the last peg is turned and in place.

Both right and left hands are tested. The other hand remains on the table.

Easier Version:

If this task is too difficult, use the simpler version: the lower row is empty (three holes). All pegs are moved one row down.

Record the time it takes to move all the pegs. Stop the timer when the last peg is in place. Both right and left hands are tested.

Figure S3. Turn pegs with right hand. Supporting hand is allowed to hold the board if necessary.


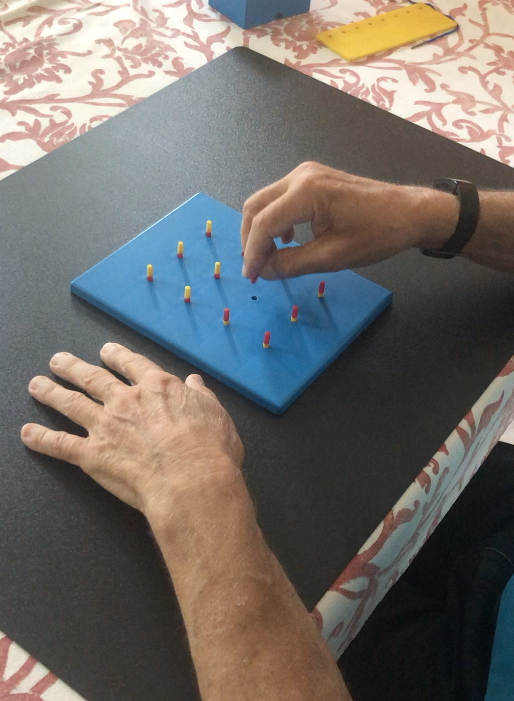


### 4. Threading Lace

Record the time taken to thread the lace through the whole board until the slack is pulled out. (See Figure S4)

See instruction manual MABC-2 age band 2 (Henderson, Sugden, Barnett 2007).

Figure S4. Threading lace through board.


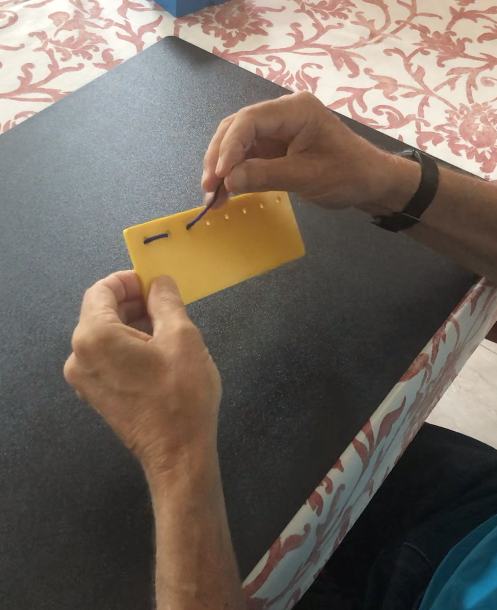


### 5. Pen and Paper Trail (Preferred Hand)

Setup: Bic Atlantic pen and smooth writing pad.

Emphasize accuracy (stay between boundaries); accuracy is more important than speed. However, if participants go too slowly, they may make more mistakes.

#### Instructions:

- Trace with pen and stay between the boundaries (see figure S5).

- If a participant goes outside the trace (count as a mistake), they should restart at the point where they went out of the trace and continue.

- Ensure a good sitting position, with the non-preferred hand stabilizing the paper at a comfortable angle.

Record time (in seconds) as well as the number of mistakes. Participants should aim not to make mistakes and then to go as fast as possible. Mistakes include traces outside the boundaries or gaps in the trace.

Figure S5. The paper trail.


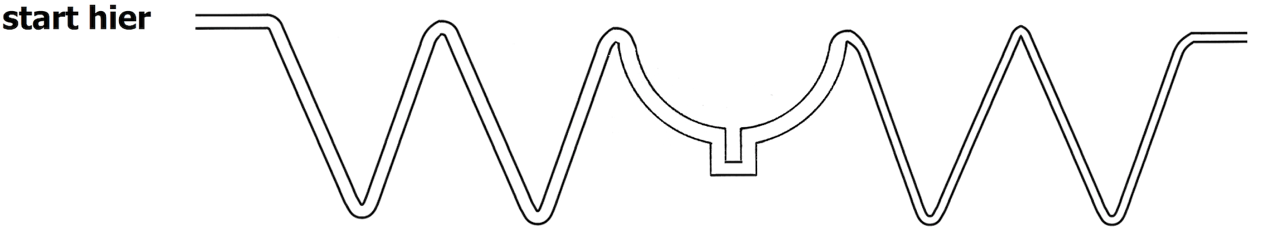


## Functional Strength

1. Sit to Stand

2. Lifting Box

3. Grip Strength (Left & Right)

### 1. Sit to Stand

See instruction manual Functional Strength Measure (Bouwien Smits-Engelsman, Wendy Aertssen 2018).

#### Equipment:

- Chair, bench, or treatment plinth at a 90-degree angle for hips and knees.

- Stopwatch/timer.

#### Measured Parameters:

Number of repetitions within 30 seconds.

#### Performance:

The patient sits with feet flat on the floor, parallel, and hip-width apart. The patient is instructed to stand up with hands clasped and arms extended forward, maintaining this position when sitting down again. When standing, knees and hips must extend to within 15 degrees of full extension.

#### Demonstration and Instructions:

The test is demonstrated and practiced for a maximum of 3 sit-to-stands.

Figure S6 A and B. Hands folded during sit (A) to stand (B)


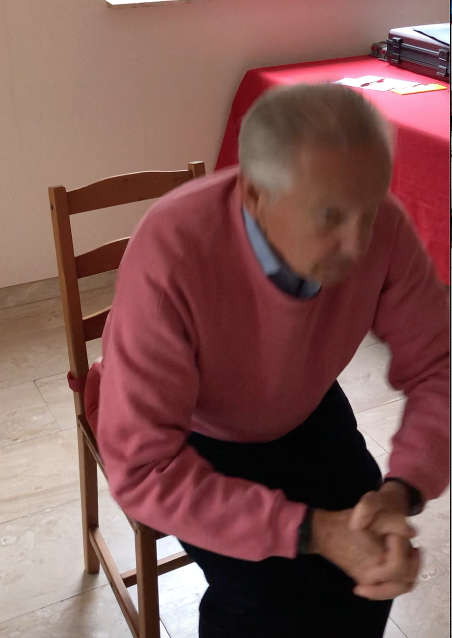

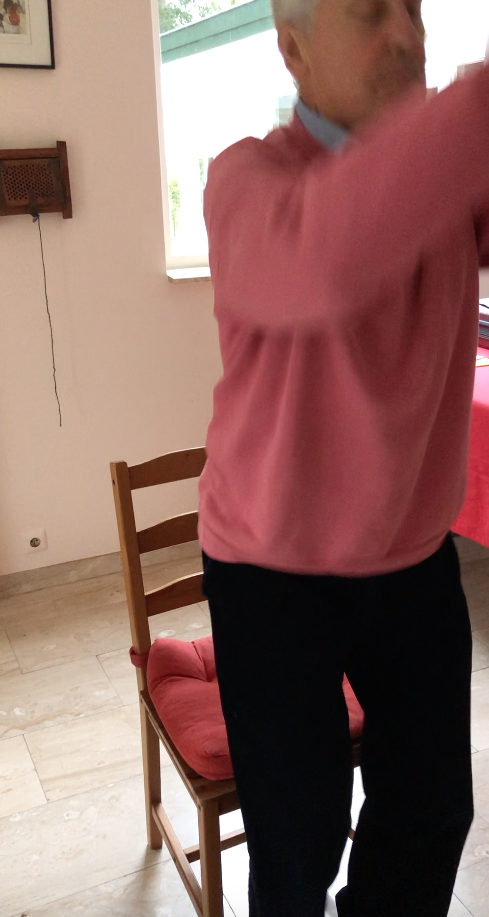


**S6B**

**S6A**

### 2. Lifting Box

See instruction manual Functional Strength Measure (Bouwien Smits-Engelsman, Wendy Aertssen 2018).

Two standard Curver boxes are used. In the “lifting box,” there are 5 kg sandbags. The second (stable) box is positioned one width of the box away from the front of the table. After the starting signal, the patient lifts the box onto the stable box and back to the table as many times as possible in 30 seconds. Outcome: number of successful trials.

Figure S7 A and B. LIfting the box from the table (A) on the box on top of the second (B)

**S7B**


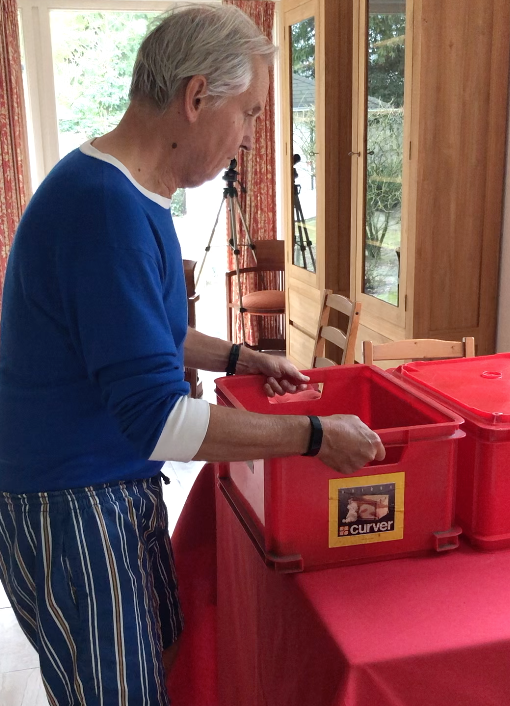

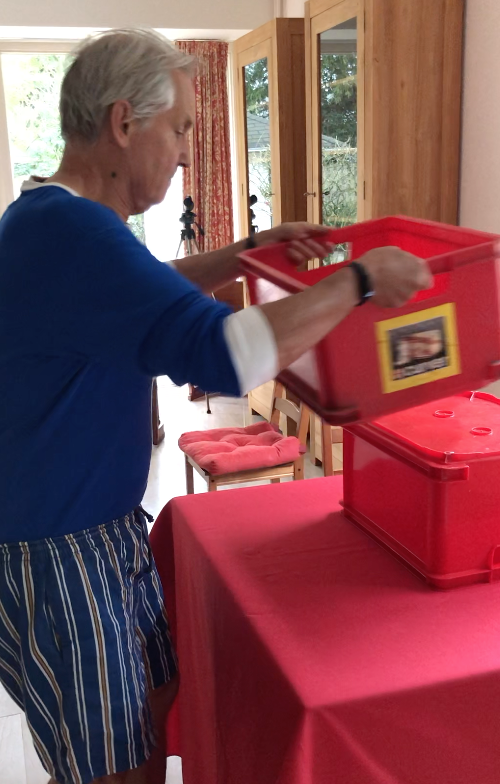


**S7A**

### 3. Grip Strength (Left & Right)

Grip strength is measured using a hand-held dynamometer.

The patient sits at the table, elbow on the table, and squeezes the dynamometer with maximum strength.

Perform two trials in pronation (Thumb on top) and two in supination (Thumb sideways) with each hand. Outcomes are recorded in kg.

## Walking with Directional Changes (Dynamic Balance, Freezing)

1. Agility Ladder, Running

2. Agility Ladder, Stepping

3. Agility Ladder, Sideways (Left & Right)

4. Rotation on Mat: Turn in Circle Clockwise and Anticlockwise

### 1. Agility Ladder, Running

See instruction manual PERF-FIT (Smits-Engelsman 2018).

#### Task:

The patient starts with both feet behind the starting line (in front of the first bar of the agility ladder). On the starting signal, the patient runs, placing one foot in each square, then runs around a bottle and back through the ladder to the end. Time ends when both feet are over the starting line and on the floor.

#### General Instructions:

“You will run as fast as possible while staying in the squares, avoiding the bars, around the bottle, and back. I’ll say ‘ready, steady, GO!’ and you start running. I’ll stop the timer when you are back over the starting line with both feet on the floor. Let me demonstrate. Did I step on any of the bars? Now you can practice. Are you ready to try it now?”

#### Scoring:

The practice trial is for warming up and ensuring understanding of the task. Time is recorded in 0.01 seconds for 2 trials. Rest time between the two trials is 15 seconds. Count and record mistakes. For each mistake made, add 0.5 seconds to the time scored for this item. A mistake is counted if:

1. The patient steps on a bar

2. Steps outside the square with one foot

3. Makes the wrong running pattern (e.g., two feet in one square or skipping a square)

4. Loses balance

Record if freezing occurs during the run or at the turning point.

Remark: Rate how stooped the walking pattern is on a 3-point scale: 1 = normal, 2 = moderate, 3 = close to tipping over.

Figure S8A (left) shows the dimensions, stepping pattern and set up of the agility ladder. S8B (right) shows start option of the running item.

**S8B**


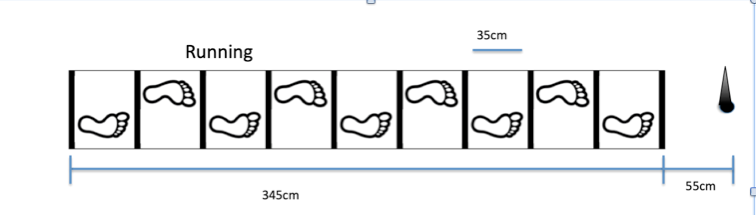

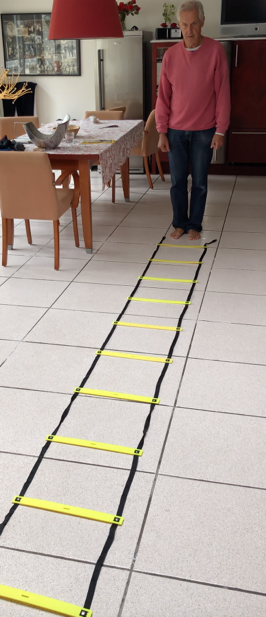


**S8A**

### 2. Agility Ladder, Stepping

S9 shows the dimensions, stepping pattern and set up of the agility ladder


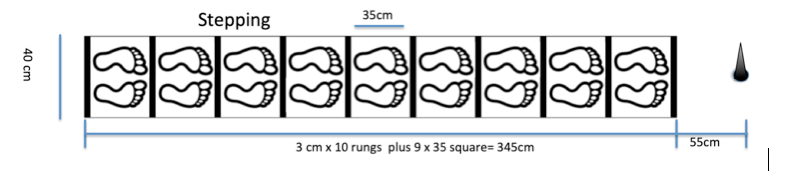


#### Equipment:

- Agility ladder

- 1500 ml pet bottle filled with water, approximately 50 cm from the last bar of the agility ladder (depending on bottle size). The total distance between the outside of the first bar and the back of the bottle should be 4 meters (See Figure S9).

#### Task:

The patient starts with both feet in front of the starting line (first bar of the agility ladder). On the starting signal, the patient runs, stepping with two feet in each square (no jumping), then runs around the bottle and back in the squares of the ladder to the end (two feet on the floor and over the starting line).

#### General Instructions:

“You will do this again, but now you must step with two feet in every square. I’ll demonstrate: one-two, one-two. Step as quickly as possible in the squares, one-two, run around the bottle, and back stepping one-two, one-two. I’ll say ‘ready, steady, GO!’ and you start stepping. I’ll stop the timer when you are back over the starting line with both feet on the floor.”

Practice is important as some individuals may initially have difficulty understanding the stepping pattern.

#### Scoring:

Time is recorded in 0.01 seconds for 2 trials. Rest time between the two trials is 15 seconds. Count and record mistakes. For each mistake made, add 0.5 seconds to the time scored for this item. A mistake is made if:

1. The patient steps on a bar

2. Steps outside the square with one foot

3. Makes the wrong stepping pattern (e.g., only one foot in the square or skipping a square)

4. Loses balance

Record if freezing occurs during the run or at the turning point.

### 3. Agility Ladder, Sideways (Left & Right)

#### Equipment:

- Agility ladder

- 1500 ml pet bottle filled with water, approximately 50 cm from the last bar of the agility ladder (depending on bottle size). The total distance between the outside of the first bar and the back of the bottle should be 4 meters.

#### Task:

The patient starts with both feet in the first square. On the starting signal, the patient steps one foot into the next square far enough for the other foot to be placed in the same square. When moving to the right, the right leg is lifted first and placed in the second square, followed by the left foot, continuing until both feet are in the last square (where the patient needs to stop). This is the starting position for the return journey to the left, beginning with the left foot. (See Figure S10)

#### Scoring:

Time is recorded in 0.01 seconds for 2 trials. Rest time between the two trials is 15 seconds. Count and record mistakes. For each mistake made, add 0.5 seconds to the time scored for this item. A mistake is made if:

1. The patient steps on a bar

2. Steps outside the square with one foot

3. Makes the wrong stepping pattern (e.g., only one foot in the square or skipping a square)

4. Loses balance

Record if freezing occurs.

Figure S10 A and S10B show the stepping pattern of the side stepping item and set up of the agility ladder.


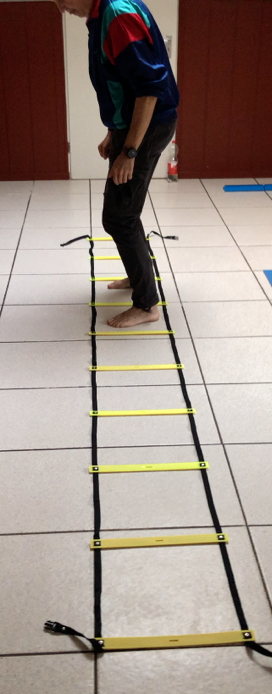

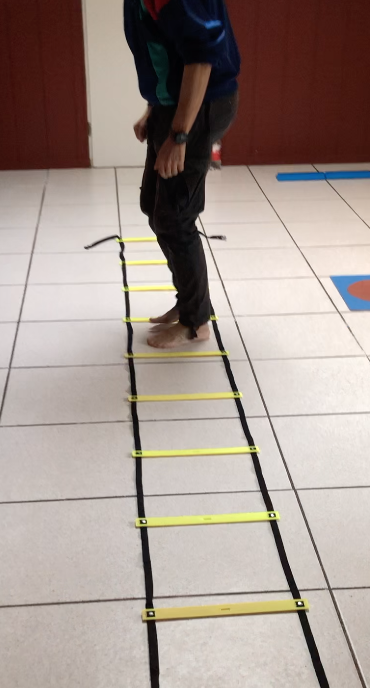


**S10B**

**S10A**

### 4. Rotation on Mat: Turn in Circle Clockwise and Anticlockwise

Start Position**:** Two feet outside the mat. Step into the circle and rotate until both feet are back on each side of the circle line, then step out again. Stop the timer when both feet are on the floor on the side of the mat. Task is done clockwise and anticlockwise. Record time in milliseconds and freezing episodes are counted as well.

Figure S11 A shows the starting position of item rotation on the mat and S11B turning in the circle.


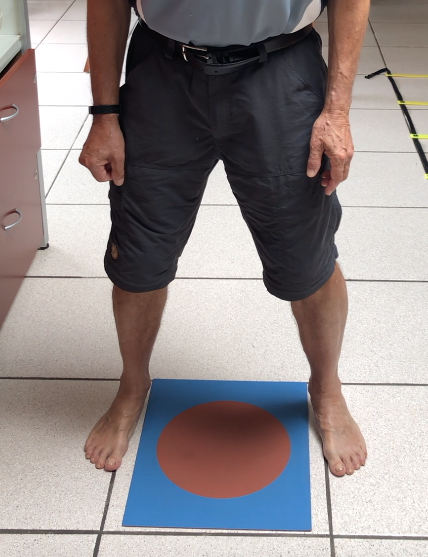

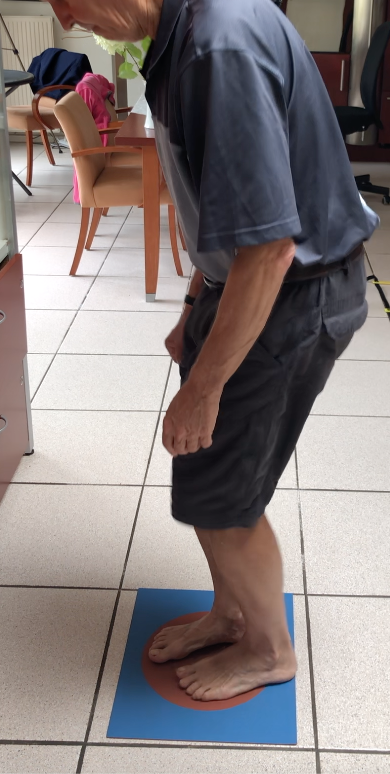


**S11B**

**S11A**

## Static Balance-Stability

### 1. Foot on foot (15 seconds max)

- Stand on right foot, placing the instep of the left foot against the big toe of the right foot. Hands on hips.

- Stand on left foot, placing the instep of the right foot against the big toe of the left foot.

2. Tandem stance (Left & Right in front) (15 seconds max) with hands on hips.

3. Knee hug (standing on Left & standing on Right) (15 seconds max). See figure S12.

Time: Record in seconds.

Figure S12. Standing while hugging the knee.


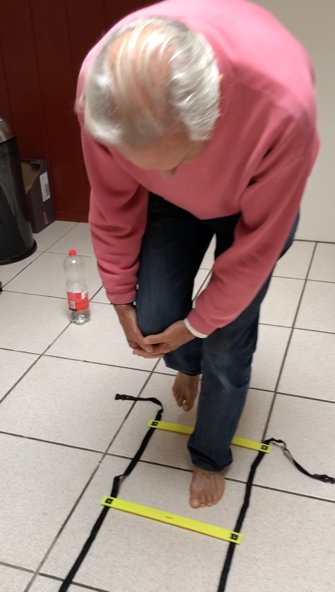


Extra ADL Activities (All items are videotaped)

1. Putting on pants (sitting and standing)

2. Putting on socks

3. Buttoning shirt

4. Turning in bed (on yoga mat, clockwise and anticlockwise)

Qualitative criteria need to be formulated.

Standardized start and end positions use the patient’s own size clothes (use the same set for repeated tests).

- a) Shorts

- b) Socks

- c) Shirt

1. Putting on Pants (Sitting and Standing)

Start Position**:**

Patient is sitting on a chair, with pants on the floor within arm’s reach.

In Standing: Patient reaches forward to pick up pants while standing, then steps into them. As soon as the pants are on, the patient claps hands to signal the end of the timer.

In Sitting: Patient first gets legs into the pants, then stands up to pull them up. As soon as the pants are on, the patient claps hands to signal the end of the timer.

Figure S13A Start position for putting on shorts, S13B in sitting and S13C in standing.

**S13C**

**S13B**

**S13A**


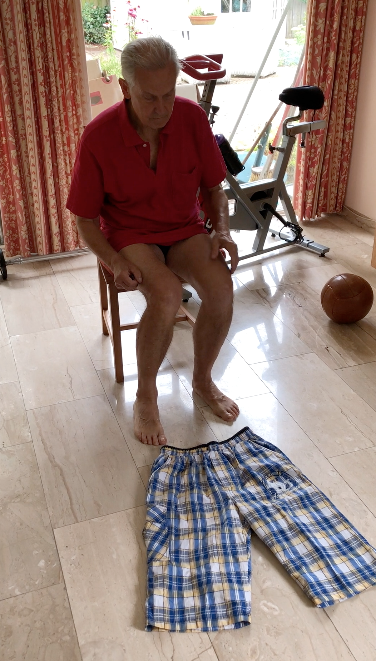

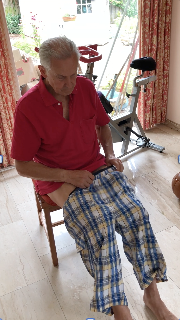

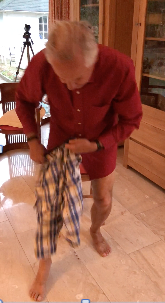


### 2. Putting on Socks

Start Position: Patient is sitting on a chair, with socks on the floor within arm’s reach (See Figure S14). The patient puts on the sock. As soon as the sock is on, the patient places the foot back on the floor to signal the end of the timer. Both feet are tested separately. Timer stops when foot with sock is put on the ground. L and R tested separately.

Figure S14A Start position for putting on socks, S14B, S14C, S14D progress putting on socks.

**S14D**

**S14B**


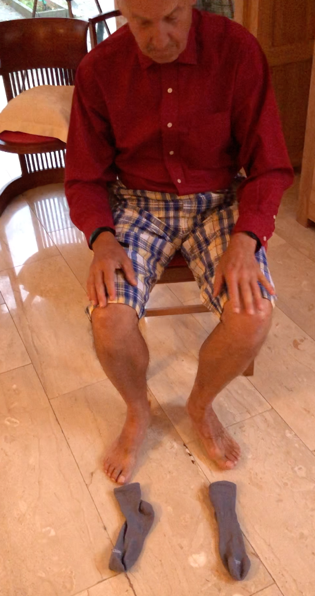

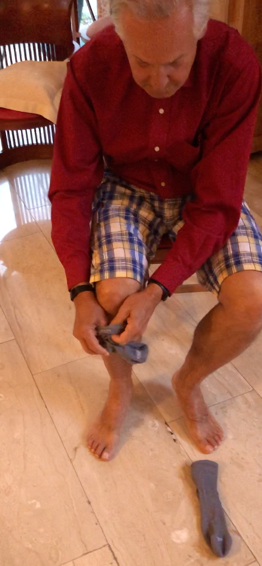

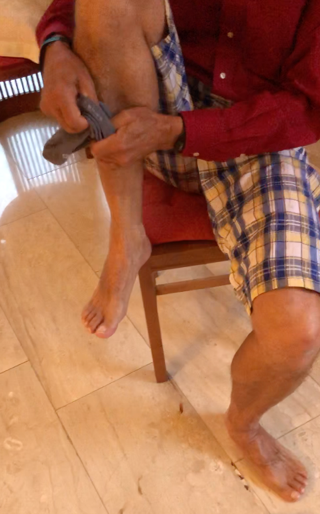

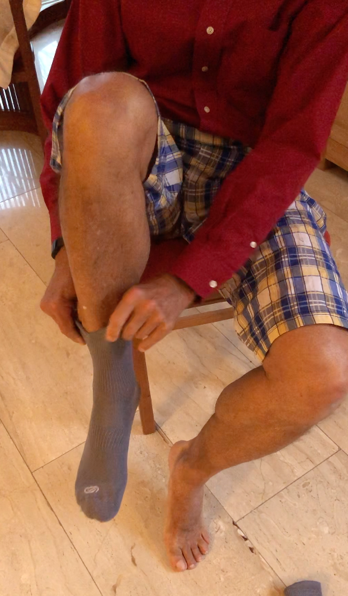


**S14C**

**S14A**

### 3. Buttoning Shirt

Use a slightly oversized shirt with buttons. At the start, the patient is standing with the shirt on but unbuttoned. Give the ready, steady, go signal. The patient buttons four buttons, and the time is recorded. If possible, also use buttons at the sleeves.

Figure S15A and S15B (top row) Buttoning shirt, S15C and S15D (bottom row) buttons on the sleeves, the one-handed task.


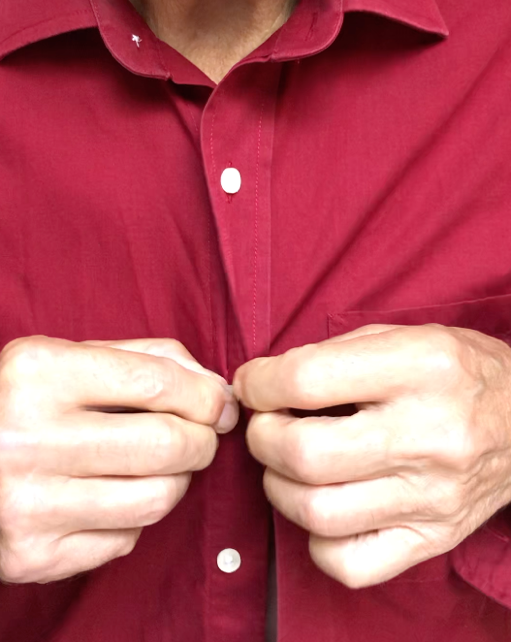

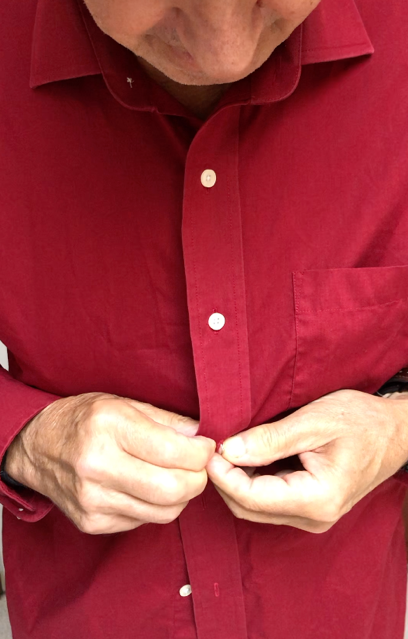


**S15B**

**S15A**


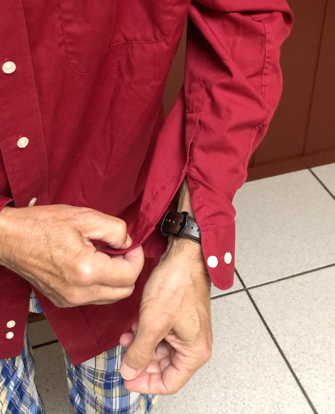

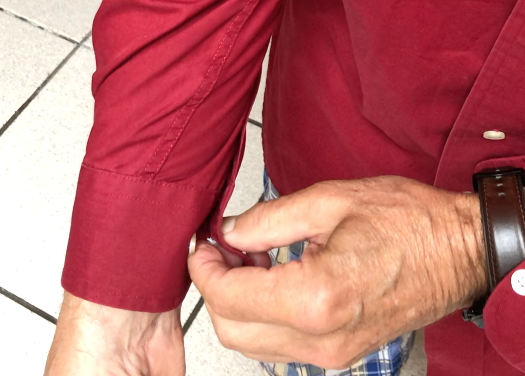


**S15D**

**S15C**

4. Turning in Bed (on Yoga Mat, Clockwise and Anticlockwise)

Start Position: Patient lies on his/her back with hands at their sides on a yoga mat (60 cm width).

On the signal, the patient turns on the mat (not allowed to go outside the boundaries)

Stop the timer when the patient is flat on their belly with hands flat on the mat next to the shoulders (W-position). Both clockwise and anticlockwise rolling is scored. Record time in seconds.

Figure S16A Shows the start position for turning on the mat, S16B, S16C, progress in turning, S16D end position.

**S16A**


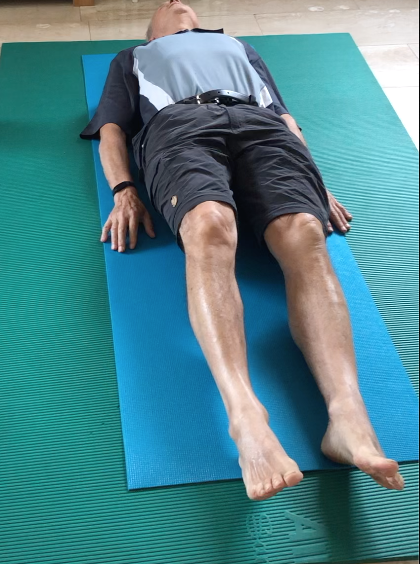

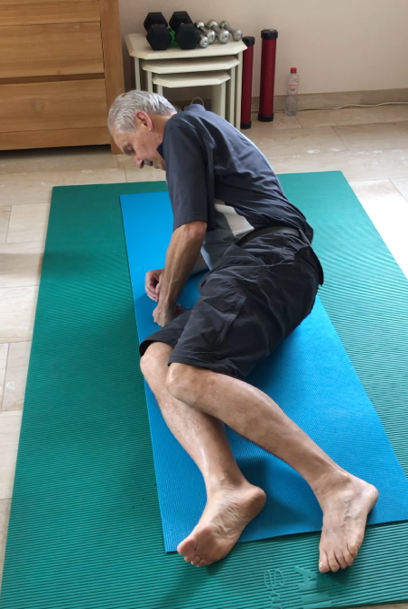

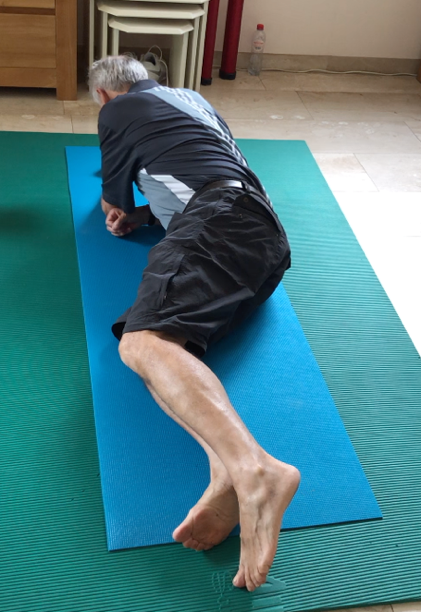

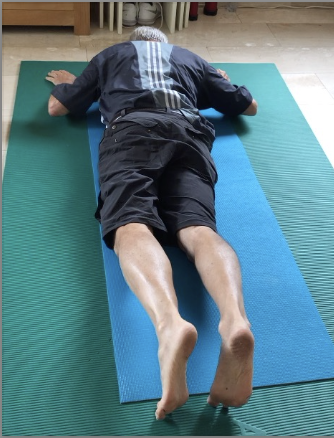


**S16C**

**S16D**

**S16B**

**16D**

#### Additional Info on score form

- Ask participants to list activities they have trouble with.

- Which other activities not tested in the PD-FUNC are you experiencing difficulties with?

- Describe the posture of the client (Posture Rating Chart).
