# Supplementary material for: Parkinson’s disease functional movement battery a comprehensive test set to evaluate of motor abilities in persons with Parkinson’s disease
Source: Sci Rep. 2025 Apr 8;15:12035. doi: 10.1038/s41598-025-96594-3 (PMC11978789; doi:10.1038/s41598-025-96594-3)
Supplement: Supplementary file 2 — Supplementary Information 2. [file 41598_2025_96594_MOESM2_ESM.pdf]

This appendix provides an overview of the longitudinal use of the PD-FUNC with the patient, JD.

The PD-FUNC test battery was applied over four years to patient JD (co-author) to monitor disease progression and medication effectiveness. Notably, dynamic balance, as measured by the agility ladder items, deteriorated in 2022, coinciding with an increase in falls and episodes of freezing (see Figure S17).

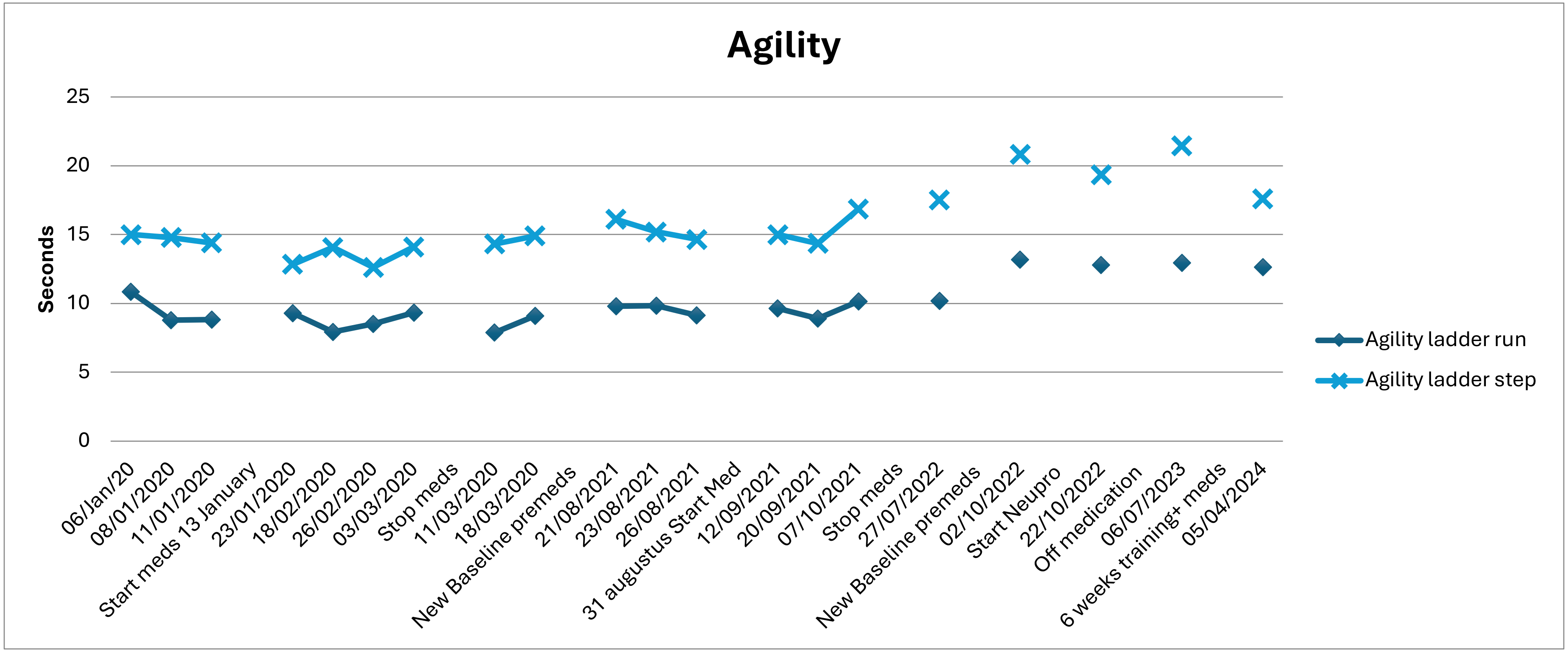

Figure S17. Patient JD was diagnosed in September 2017. The first three tests in 2020 established a baseline and evaluated the possible learning effect. A levodopa trial commenced on January 13, 2020 (6 weeks, levodopa 50 mg three times daily), but showed no improvement in running or stepping; hence, medication was discontinued after 6 weeks (March 3, 2020).
A second levodopa trial (50 mg levodopa with 12.5 mg benserazide, three times daily) began on August 31, 2021, again with no impact on locomotion, leading to discontinuation after six weeks (October 7, 2021).

In 2022, worsening gait performance and increased freezing prompted a trial with a dopamine agonist (Rotigotine transdermal patches), which was halted due to side effects and lack of efficacy. In early 2024, a six-week intense training program was initiated alongside a regimen of 100 mg levodopa three times daily. Subsequent measurements indicated a reduction in time required for stepping on the agility ladder, reflecting improved dynamic balance.

Furthermore, JD's longitudinal assessment demonstrated the potential of the PD-FUNC battery to evaluate the differential effects of medications. As shown in Figure S18, performance on the pegboard test (right hand, more affected side) improved significantly after levodopa administration, while no positive change was observed in gait performance on the agility ladder.

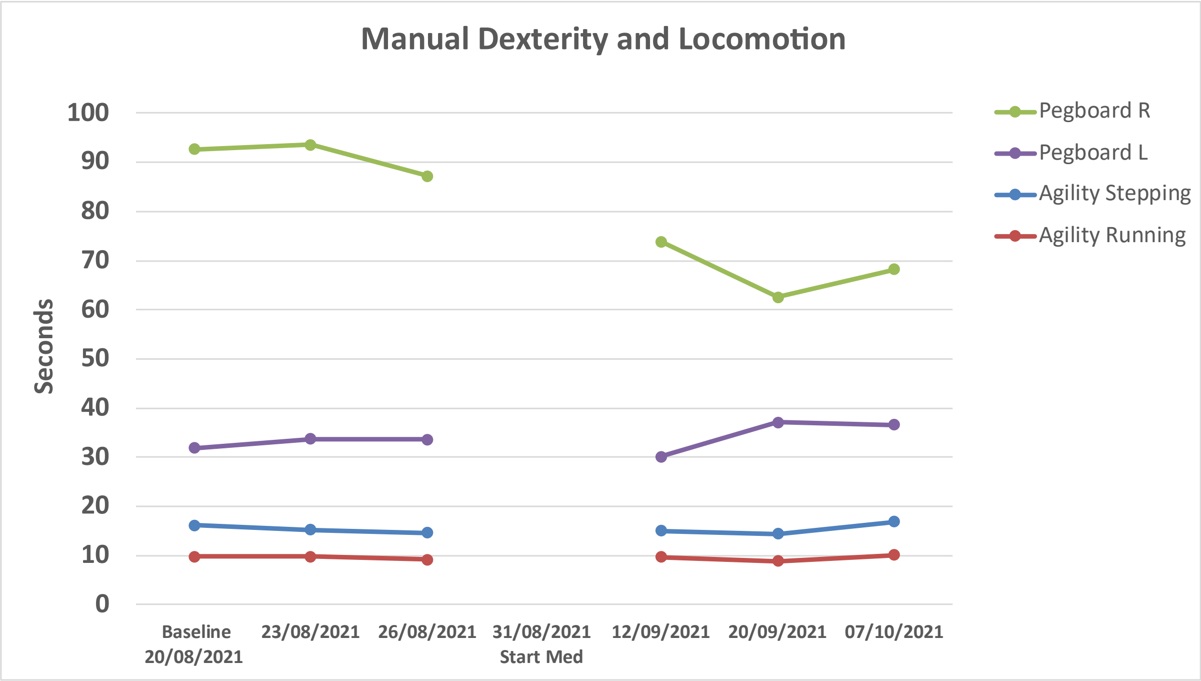

Figure S18. Pre- and post-test results on four items before and after initiating the levodopa regimen (50 mg levodopa with 12.5 mg Benserazide, three times daily). The right hand, being more affected, showed some response to medication, whereas no improvement in locomotion was noted.
